# Exposure to Ambient Air Fine Particulate Matter Prevents VEGF-Induced Mobilization of Endothelial Progenitor Cells from the Bone Marrow

**DOI:** 10.1289/ehp.1104206

**Published:** 2012-03-14

**Authors:** Petra Haberzettl, Jongmin Lee, Dheeraj Duggineni, James McCracken, Duane Bolanowski, Timothy E. O’Toole, Aruni Bhatnagar, Daniel J. Conklin

**Affiliations:** Diabetes and Obesity Center, University of Louisville, Louisville, Kentucky, USA

**Keywords:** air pollution, AMD3100, CAP, cardiovascular disease, environmental cardiology, flow cytometry, PM_2.5_, SCF, stem cells, VEGF

## Abstract

Background: Exposure to ambient fine particulate matter air pollution (PM_2.5_; < 2.5 µm in aerodynamic diameter) induces endothelial dysfunction and increases the risk for cardiovascular disease. Endothelial progenitor cells (EPCs) contribute to postnatal endothelial repair and regeneration. In humans and mice, EPC levels are decreased upon exposure to elevated levels of PM_2.5_.

Objective: We examined the mechanism by which PM_2.5_ exposure suppresses circulating levels of EPCs.

Methods: Mice were exposed to HEPA-filtered air or concentrated ambient fine particulate matter (CAP, 30–100 µg/m^3^) from downtown Louisville (Kentucky) air, and progenitor cells from peripheral blood or bone marrow were analyzed by flow cytometry or by culture *ex vivo*.

Results: Exposure of the mice to CAP (6 hr/day) for 4–30 days progressively decreased circulating levels of EPCs positive for both Flk-1 and Sca-1 (Flk-1^+^/Sca-1^+^) without affecting stem cells positive for Sca-1 alone (Sca-1^+^). After 9 days of exposure, a 7-day exposure-free period led to complete recovery of the circulating levels of Flk-1^+^/Sca-1^+^ cells. CAP exposure decreased circulating levels of EPCs independent of apoptosis while simultaneously increasing Flk-1^+^/Sca-1^+^ cells in the bone marrow. We observed no change in tissue deposition of these cells. CAP exposure suppressed vascular endothelial growth factor (VEGF)-induced Akt and endothelial nitric oxide synthase (eNOS) phosphorylation in the aorta, and it prevented VEGF/AMD3100-induced mobilization of Flk-1^+^/Sca-1^+^ cells into the peripheral blood. Treatment with stem cell factor/AMD3100 led to a greater increase in circulating Flk-1^+^/Sca-1^+^ cells in CAP-exposed mice than in mice breathing filtered air.

Conclusion: Exposure to PM_2.5_ increases EPC levels in the bone marrow by preventing their mobilization to the peripheral blood via inhibition of signaling events triggered by VEGF-receptor stimulation that are upstream of c-kit activation. Suppression of EPC mobilization by PM_2.5_ could induce deficits in vascular repair or regeneration.

Extensive epidemiological evidence indicates a strong and positive correlation between exposure to airborne particulate matter (PM) and cardiovascular morbidity and mortality (Bhatnagar 2006; Brook et al. 2010). Short-term exposure to PM is associated with an increase in the risk of myocardial infarction, arrhythmias, endothelial dysfunction, stroke, and thrombosis, whereas chronic exposures increase the risk of mortality due to ischemic heart disease, arrhythmias, and heart failure. Nevertheless, the mechanisms by which PM exposure elevates cardiovascular disease (CVD) risk remain unclear.

Because PM exposure affects several aspects of cardiovascular disease, it is likely that it alters cardiovascular processes that are common to several manifestations of CVD. One such unifying mechanism that could contribute to multiple aspects of CVD is endothelial dysfunction. Changes in endothelial reactivity affect not only blood pressure regulation but also atherogenesis, heart failure, and arrhythmogenesis (Landmesser et al. 2004). Thus, PM-induced endothelial dysfunction could potentially explain several cardiovascular effects of PM exposure. Data from both human and animal studies indicate that changes in endothelial function are early and sensitive outcomes of PM exposure (Brook et al. 2009; Courtois et al. 2008).

Recent advances in vascular biology suggest that endothelial health and function are in part maintained by a replenishing pool of progenitor cells that reside mostly in the bone marrow. Upon tissue injury, endothelial progenitor cells (EPCs) are recruited to the site of damage where they participate in angiogenesis by differentiating into mature endothelial cells or by paracrine stimulation of existing cells (Hristov and Weber 2009). Levels of circulating EPCs are a sensitive index of endothelial health and are inversely correlated with CVD risk (Hill et al. 2003; Steinmetz et al. 2010). Unhealthy lifestyle factors, smoking (Yue et al. 2010), or acrolein exposure (Wheat et al. 2011) suppress EPC levels, indicating that EPC depletion may be a significant contributory factor linking pollutant exposure to CVD risk. Indeed, our recent studies show that short-term exposure to PM decreases circulating levels of EPCs in humans and mice (O’Toole et al. 2010). Nevertheless, the mechanisms by which PM exposure suppresses EPC levels are not known; it is unclear whether PM depletes EPCs by triggering cell death, stimulating the recruitment of these cells to injured tissue, or preventing their mobilization from the bone marrow. Accordingly, the present study was designed to investigate the nature of the EPC defect induced by PM_2.5_ (particulate matter < 2.5 µm in aerodynamic diameter) and to identify specific processes involved in EPC mobilization and homing that are affected by PM_2.5_ exposure.

## Materials and Methods

*PM_2.5_ and concentrated ambient particle (CAP) analysis.* Ambient PM_2.5_ was collected between June 2009 and December 2010 at the Medical and Dental Research building (7th floor) at the Health Science Center, University of Louisville (Louisville, KY; 38°15´15˝N/85°45´33˝W). Ambient PM_2.5_ was concentrated using a versatile aerosol concentration enrichment system (VACES). A single-stage stainless steel filter holder with a teflon filter (47 mm diameter) was used to collect ambient PM_2.5_ or CAP at 10 L/min or 1 L/min air flow rate, respectively. Collected PM mass concentrations were determined gravimetrically using a microbalance in a room with controlled temperature and humidity. Analysis of ambient PM_2.5_ and CAP filters indicated a VACES PM_2.5_ concentration factor of 2.7- to 9.3-fold. CAP concentrations varied between experiments as a function of ambient PM_2.5_ levels and meteorological conditions, including temperature and relative humidity [see Supplemental Material, Table 1 (http://dx.doi.org/10.1289/ehp.1104206)]. In addition, a nephelometer was used to measure real-time CAP mass concentration and mass median diameter.

For analysis of ambient PM_2.5_ or CAP chemical composition and physical properties, particles were collected using different filter types. The organic carbon and elemental carbon compositions were analyzed by a thermal-optical analysis technique. Standard U.S. Environmental Protection Agency (EPA) methods were used to analyze sulfate, nitrate, and ammonium ion levels. Elemental analysis was conducted by X-ray fluorescence spectrometry.

Particle size distributions of ambient PM_2.5_ and CAP were analyzed as geometric particle number distributions using a filter-based particle size analyzer (Malvern Mastersizer 2000; Malvern Instrument Ltd., Worcestershire, UK). The relative refractive index on the applied particle size analyzer was 1.16. To better characterize the relationship between CAP exposure and EPC suppression, exposure duration and cumulative CAP load were regressed against EPC level. The estimate of CAP load was calculated as follows: CAP (micrograms per cubic meter) × chamber flow (cubic meters per minute) × exposure time (minutes). We estimated lung deposition of PM for each specific exposure using mouse tidal volume, breathing frequency, and an estimated deposition fraction. See Supplemental Material, pp. 3–4 (http://dx.doi.org/10.1289/ehp.1104206) for additional details.

*Mice and exposures.* Mice were treated humanely and with regard for alleviation of suffering according to the *Guiding Principles in the Care and Use of Animals* (American Physiological Society 2012). All protocols were approved by the University of Louisville Institutional Animal Care and Use Committee. Male C57BL/6J mice (8–12 weeks of age; Jackson Laboratory, Bar Harbor, ME) were exposed to HEPA-filtered air or concentrated ambient PM_2.5_ (CAP).

*Progenitor cell mobilization.* To mobilize progenitor cells, mice were injected subcutaneously with recombinant murine VEGF_165_ (vascular endothelial growth factor) in saline (0.1 mL, 100 µg/kg/day; Peprotech, Inc., Rocky Hill, NJ) daily for 4 consecutive days (Pitchford et al. 2009). In another group, mice were injected subcutaneously with a single bolus of stem cell factor (SCF; 0.1 mL, 200 µg/kg; Peprotech, Inc.). In both protocols, mice received the CXCR4 (chemokine X receptor type 4) antagonist AMD3100 (5 mg/kg, intraperitoneal injection, 0.1 mL in saline; Sigma-Aldrich, St. Louis, MO) or saline alone (0.1 mL; control). Blood, bone marrow, and tissues were harvested 1 hr after AMD3100 or saline injection. See Supplemental Material (http://dx.doi.org/10.1289/ehp.1104206) for additional details.

*Isolated aorta studies.* Thoracic aortas were isolated for assessment of either VEGF signaling or vascular reactivity as described previously (Wheat et al. 2011; Conklin et al. 2009) with some modifications.

*VEGF signaling.* Thoracic aortas from distal of the aortic arch to the diaphragm were isolated from mice exposed for 9 days to air or CAP, cleaned in cold phosphate-buffered saline (PBS), and then placed in autologous plasma for 1 hr at 37°C prior to addition of saline (vehicle) or VEGF (20 ng/mL) for 15 min. After incubation, aortas were snap frozen in liquid nitrogen and stored at –80°C before use for Western blotting.

*Vascular reactivity.* Briefly, one 3–4-mm aortic ring per mouse was hung on stainless steel hooks in 15-mL water-jacketed organ baths in physiological salt solution bubbled with 95% O_2_ and 5% CO_2_ at 37°C. Aortic rings were contracted with 100 mM potassium solution twice and reequilibrated to approximately 1 g over 2 hr before measuring endothelial function. To measure endothelium-dependent relaxation, phenylephrine-precontracted aortas were relaxed with cumulative concentrations of acetylcholine. To measure endothelium-independent relaxation, aortas were precontracted with either 100 mM potassium (9-day study) or with U46619 (thromboxane A_2_ analog, 0.1 μM; 30-day study) and then relaxed with cumulative concentrations of sodium nitroprusside. Relaxation was calculated as a percentage reduction of agonist-induced tension. For additional details, see Supplemental Material, pp. 6–7 (http://dx.doi.org/10.1289/ehp.1104206).

*Flow cytometry.* Mononuclear cells from peripheral blood or bone marrow were separated by Ficoll gradient centrifugation and immunolabeled with PE-anti-Sca-1 (phycoerythrin-conjugated stem cell antigen-1) and APC-anti-Flk-1 [allophycocyanin-conjugated fetal liver kinase-1 (VEGFR2, vascular endothelial growth factor receptor 2), 1 µg; BD BioSciences, San Jose, CA]. The number of cells positive for both Flk-1 and Sca-1 (Flk-1^+^/Sca-1^+^) and those positive for Sca-1 alone (Sca-1^+^) were measured by flow cytometry using an LSRII flow cytometer (BD BioSciences) and analyzed with FlowJo v8 software (Treestar Inc., Ashland, OR). For measuring apoptosis, the mononuclear cell population was incubated with Annexin Binding Buffer (100 µL for 15 min at room temperature; Annexin Kit; eBioscience, San Diego, CA) and labeled with Flk-1 and Sca-1 antibodies. To detect necrosis, the cells were labeled with 7-AAD (7-aminoactinomycin D; 5 µL; eBioscience) and analyzed by flow cytometry. After select exposures, 100 µL whole blood was used for complete blood count analysis (CBC; Hemavet 500; Coulter Counter, Oxford, CT).

*Cell culture.* Mononuclear cells from blood (1 × 10^5^ to 4 × 10^5^ cells) and Ficoll-separated bone marrow (8 × 10^5^ cells) were cultured for 4 or 7 days, respectively. After incubation with DiI-acLDL (1,1´-dioctadecyl-3,3,3´,3´-tetramethylindocarbocyanine perchlorate-acetylated low density lipoprotein, 2.4 µg/mL; Molecular Probes, Invitrogen, Carlsbad, CA) for 3 hr (37°C, 5% CO_2_), blood cells were fixed in 4% paraformaldehyde/PBS, pH 7.4 (10 min at room temperature) and incubated with FITC-UE-lectin (fluorescein isothiocyanate–*Ulex europaeus*-lectin; 50 µg/mL, 30 min, 37°C; Sigma-Aldrich). To label bone marrow-derived cells (BMDCs), fixed cells were incubated with FITC-Sca-1 (1:25; BD BioSciences) and APC-Flk-1 (1:15, BD BioSciences) antibodies (1 hr at room temperature). Slides were mounted with DAPI (4´,6-diamidino-2-phenylindole)-containing Slow Fade® Gold anti-fade reagent (Invitrogen); DiI-acLDL^+^/FITC-UE-lectin^+^ or FITC-Sca-1^+^/APC-Flk-1^+^ cells were counted in 10 random microscopic fields. For the tube-forming assay, 4 × 10^4^ BMDC were seeded into Matrigel™-coated plates and microscopic images were acquired after 24 hr in culture.

*Western blot analyses.* Proteins in the tissue lysates of heart, lung, spleen, and pulverized aortas [see Supplemental Material, p. 7 (http://dx.doi.org/10.1289/ehp.1104206)] were separated by SDS-PAGE, transferred to PVDF membranes and probed with antibodies against VEGFR-2, phosphorylated (phospho)-Akt (Ser473), Akt, phospho-eNOS (phosphorylated endothelial nitric oxide synthase; Ser1177), and eNOS (1:1,000), all from Cell Signaling Technology (Danvers, MA) or Id-1 (inhibitor of DNA binding 1; 1:1,000, Proteintech, IL), as described previously (Wheat et al. 2011). See Supplemental Material, p. 7 (http://dx.doi.org/10.1289/ehp.1104206) for additional details.

*Statistical analysis:* Data presented are mean ± SE. We used Student’s *t-*test for two-group comparison and one-way analysis of variance (Bonferroni post hoc test) for multiple group comparison. Statistical significance was accepted at *p* < 0.05.

## Results

**Figure 1:**
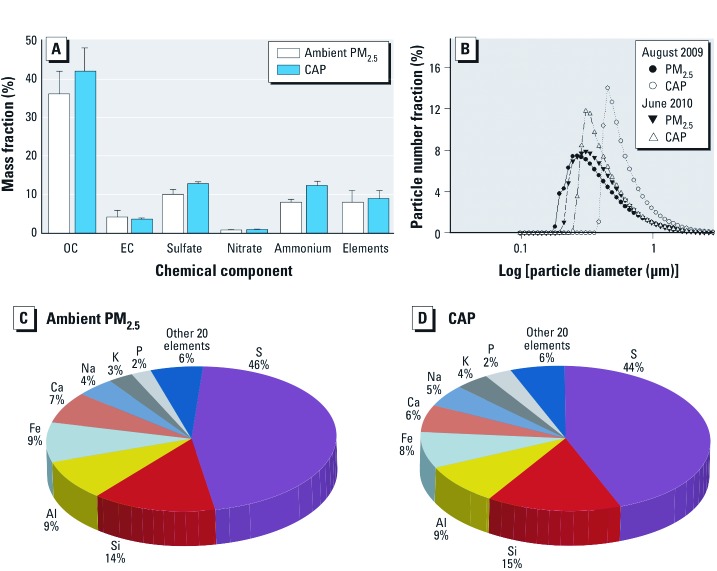
Chemical and physical characterization of ambient PM_2.5_ and CAP in Louisville, Kentucky. (*A*) Overall chemical composition of PM_2.5_ and CAP. (*B*) Overall particle size distributions as analyzed on the basis of equivalent number diameter. (*C,D*) Elemental composition of ambient PM_2.5_ (*C*) and VACES-collected CAP (*D*) from downtown Louisville as an average of several exposures performed between 2009 and 2010 [see Supplemental Material, Table 2 (http://dx.doi.org/10.1289/ehp.1104206)]. Abbreviations: Al, aluminum; Ca, calcium; Fe, iron; K, potassium; Na, sodium; P, phosphorus; S, sulfur; Si, silicon.

*Louisville PM_2.5_ and VACES characterization.* Ambient PM_2.5_ was concentrated using a VACES, and the CAP concentration varied between experiments as a function of ambient PM_2.5_ levels and meteorological conditions [e.g., temperature and relative humidity; see Supplemental Material, Table 1 (http://dx.doi.org/10.1289/ehp.1104206)]. We observed little difference between ambient PM_2.5_ and chemical composition and physical properties of CAP (organic carbon, elemental carbon, sulfate, nitrate, ammonium, elements, particle size distribution; Figure 1). For example, ambient PM_2.5_ and CAP showed no significant difference of mass fraction (as a percentage) in chemical or elemental composition (Figure 1A,C; see also Supplemental Material, Table 2). The number median diameter of ambient PM_2.5_ (0.33 µm for August 2009; 0.36 µm for June 2010) was similar to CAP number median diameter (0.53 µm, August 2009; 0.38 µm, June 2010) (Figure 1B). These data indicate the VACES concentrated ambient PM_2.5_ but did not alter its specific properties.

**Figure 2:**
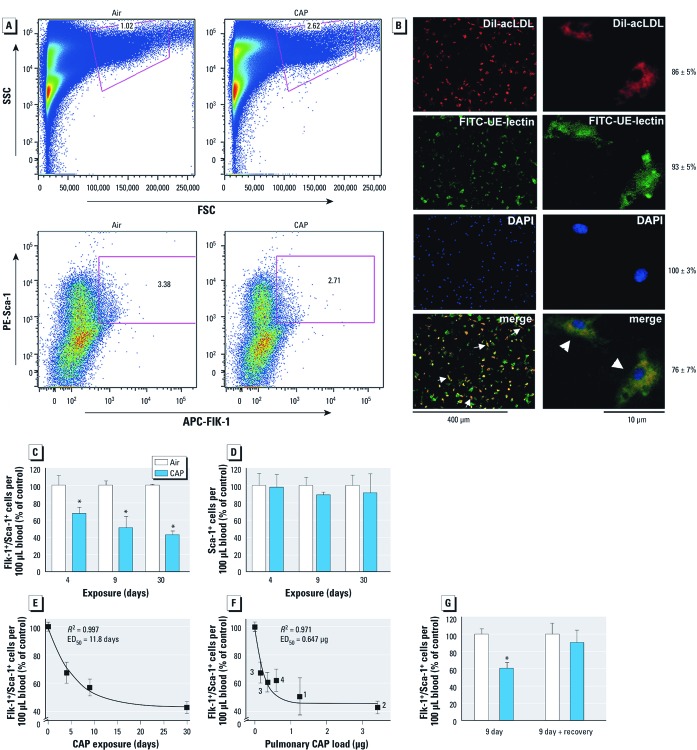
CAP exposure decreases circulating levels of Flk‑1^+^/Sca‑1^+^ cells. (*A*) Representative flow cytometry plots of side scatter (SSC) and forward scatter (FSC) or PE-Sca‑1^+^/APC-Flk‑1^+^ cells isolated from mice exposed for 9 days to either filtered air or CAP. Numbers within the plots indicate the percentage of PE-Sca‑1^+^/APC-Flk‑1^+^ cells within the lymphocytic gate. (*B*) Fluorescence images (left, 10×; right, 40×) of blood-derived DiI‑acLDL^+^/FITC-UE-lectin^+^ cells isolated from unexposed animals and grown for 4 days on fibronectin-coated plates. Merged images show DiI-acLDL^+^/FITC-UE-lectin^+^-cells in orange (right) with DAPI as a nuclear marker; the numbers of DiI-acLDL^+^, FITC-UE-lectin^+^, and DiI-acLDL^+^/FITC-UE-lectin^+^ cells are presented as the mean ± SE percentage of total cells (identified by DAPI staining) counted in five random microscopic fields. (*C,D*) Change in Flk‑1^+^/Sca‑1^+^ (*C*) and Sca‑1^+^ (*D*) cell populations after CAP exposure for 4, 9, or 30 days, presented as a percentage of the air control. (*E,F*) Changes in Flk‑1^+^/Sca‑1^+^ cells presented as a percentage of the air control and plotted as (*E*) a function of exposure duration (4-day: air, 145.5 ± 16.9 EPCs/100 μL; CAP, 98.1 ± 10.8 EPCs/100 μL; 9-day: air, 321.6 ± 25.5 EPCs/100 μL; CAP, 165.2 ± 44.1 EPCs/100 μL; 30-day: air, 268.4 ± 51.6 EPCs/100 μL; CAP, 129.2 ± 13.4 EPCs/100 μL) and (*F*) cumulative pulmonary CAP load (µg); data are shown as discrete points, and the curve is a best fit of a first-order exponential equation [(*y* = *y*_0 _+ *a*_1_exp(–*x/t*_1_)] to the data. In *F*, numbers 1–4 refer to specific CAP exposures shown in Supplemental Material, Table 1 (http://dx.doi.org/10.1289/ehp.1104206). (*G*) Circulating Flk‑1^+^/Sca‑1^+^ cells (percentage of air control) in mice exposed for 9 days to air or CAP without and with 7 days of recovery. In *C–G*, data are mean ± SE (*n* = 4–6). *p < 0.05 compared with air control.

*CAP exposure decreases the levels of circulating EPC.* Our previous study (O’Toole et al. 2010) showed that 9-day CAP exposure decreases circulating levels of EPCs in mice; however, we did not examine either the nature or the mechanism of this response. Hence, to understand the effects of CAP on EPCs, we first examined the time course and reversibility of the response. EPC levels in the blood were measured by counting events positive for endothelial (Flk-1) and stem cell (Sca-1) antigens using flow cytometry (Figure 2A). Flk-1^+^/Sca-1^+^ cells were gated in the lymphocytic population as indicated in the representative flow cytometry plots of side scatter and forward scatter (Figure 2A, top). The number of cells positive for both PE-Sca-1 and APC-Flk-1 (PE-Sca-1^+^/APC-Flk-1^+^) was counted from plots, and the percentage of total gated cells isolated from mice exposed for 9 days to either HEPA-filtered air or CAP showed fewer EPCs following CAP exposure (Figure 2A, bottom). For additional characterization, blood cells were cultured for 4 days (Figure 2B) and uptake of DiI-acLDL and binding to FITC-UE-lectin were analyzed. Microscopic images showed the outgrowth of DiI-acLDL^+^/FITC-UE-lectin^+^ spindle-shaped cells, as indicated by arrow in the merged images. Most of these cells were positive for both endothelial cell–specific markers (76 ± 7%; Figure 2B, merged images). Previous analysis shows that these cells are 3–5 µm in diameter and are positive for Id-1, CXCR4, and several other progenitor and lymphocytic cell markers (Wheat et al. 2011).

To examine whether PM_2.5_ exposure leads to a progressive decrease in EPC levels, we exposed mice to CAP (6 hr/day) for 4, 9, or 30 days. As shown in Figure 2C, CAP exposure led to a progressive decrease in the number of circulating Flk-1^+^/Sca-1^+^ cells; however, we observed no significant changes in the levels of Sca-1^+^ cells (Figure 2D). Complete blood count (CBC) analyses showed no effect of CAP exposure on the numbers of leukocytes or red blood cells [see Supplemental Material, Table 3 (http://dx.doi.org/10.1289/ehp.1104206)]. Time-course analysis revealed a nonlinear relationship between dose and EPC level. A steep decline in EPC levels was observed within 4 days of CAP exposure (33% decrease; *p* < 0.05); whereas longer exposures were associated with a shallower response. The half-maximal response duration (ED_50_) calculated from a first-order exponential equation was approximately 12 days (Figure 2E). The dose–response relationship was nonlinear with an ED_50_ value of < 1 µg cumulative pulmonary CAP load (Figure 2F). Suppression of circulating EPC levels by CAP was reversible. After 9 days of exposure, a 7-day exposure-free period led to complete recovery of circulating Flk-1^+^/Sca-1^+^ cells (Figure 2G). These observations indicate that exposure to CAP results in a rapid, specific, and reversible decrease in circulating EPC levels at low cumulative doses of CAP exposure.

*CAP exposure does not induce EPC death or tissue deposition.* CAP-induced depletion of circulating EPCs could be due to an increase in cell death (apoptosis/necrosis), enhanced recruitment/homing to sites of injury, or a decrease in mobilization from bone marrow. Hence, we tested each of these possibilities. To determine whether CAP-induced EPC depletion was due to an increase in EPC death, we examined apoptosis and necrosis in the circulating Flk-1^+^/Sca-1^+^ cell population by flow cytometry. Exposure to CAP depleted circulating Flk-1^+^/Sca-1^+^ cells; however, we did not observe an increase in markers of apoptosis (Annexin-V^+^) or necrosis (7AAD^+^) compared with exposure to filtered air [see Supplemental Material, Figure 1A,B (http://dx.doi.org/10.1289/ehp.1104206)]. These results suggest that EPC depletion in CAP-exposed mice is not due to an increase in cell death.

To test the possibility that EPCs are depleted due to increased recruitment to sites of injury (e.g., lung) or other stem cell niches (e.g., spleen), we measured the abundance of the EPC-specific protein Id-1 (Mellick et al. 2010) by Western blot analysis. Immunoblots of lysates prepared from lung, aorta, heart, and spleen showed no differences in the abundance of Id-1 protein in these organs after 9 days of exposure to air or CAP [see Supplemental Material, Figure 1C (http://dx.doi.org/10.1289/ehp.1104206)]. Thus, EPC depletion in CAP-exposed mice could not be attributed to an increase in EPC recruitment to sites of injury or other stem cell niches. Hence, we studied the effects of CAP exposure on EPC mobilization.

**Figure 3:**
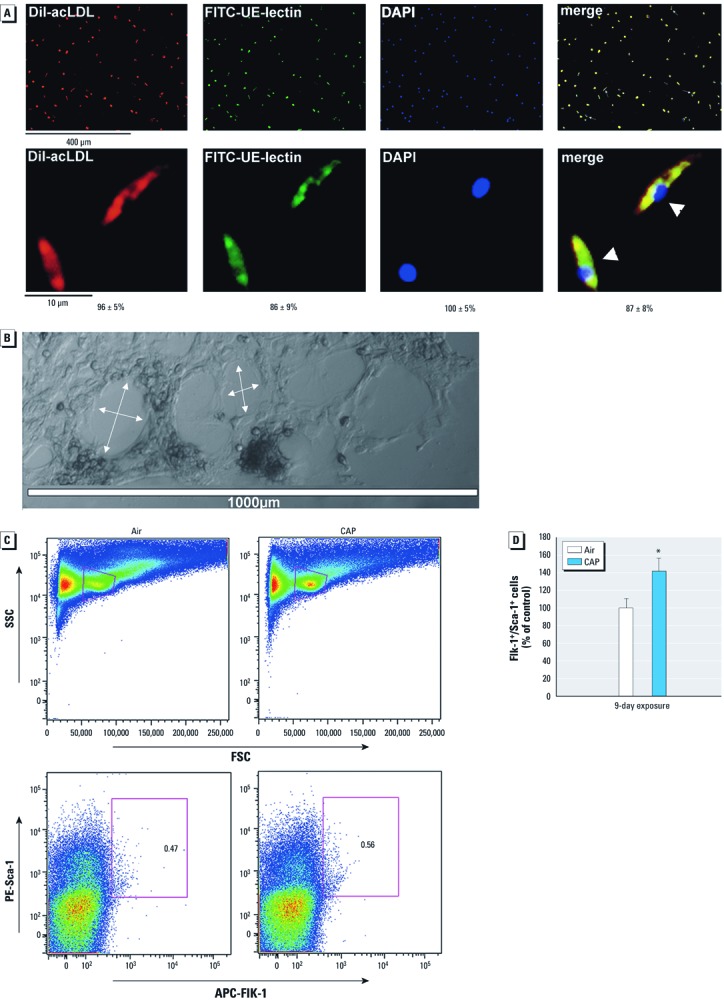
CAP exposure increases bone marrow resident Flk‑1^+^/Sca‑1^+^ cells. (*A*) Fluorescence images (top, 10×; bottom, 40×) of bone marrow-derived DiI-acLDL^+^/FITC-UE-lectin^+^ cells isolated from unexposed animals and cultured for 7 days. Merged images show DiI-acLDL^+^/FITC-UE-lectin^+^ cells in orange (right) with DAPI as a nuclear marker. The numbers of DiI‑acLDL^+^, FITC-UE-lectin^+^, and DiI-acLDL^+^/FITC-UE‑lectin^+^ cells were counted in five random microscopic fields, and the numbers of cells are presented as the mean ± SE percentage of total cells (identified by DAPI staining). (*B*) Tube formation (arrows) by EPCs isolated from bone marrow, cultured for 10 days, and then seeded on Matrigel™. (*C*) Representative flow cytometry plots of side scatter (SSC) and forward scatter (FSC) or PE-Sca‑1^+^/APC-Flk‑1^+^ fluorescence in cells isolated from bone marrow of mice exposed for 9 days to air or CAP. Numbers within the plots indicate the percentage of PE-Sca‑1^+^/APC-Flk‑1^+^ cells within the lymphocytic gate. (*D*) Changes in Flk‑1^+^/Sca‑1^+^ cells in bone marrow from mice exposed for 9 days to air or CAP (mean ± SE percent of control; *n* = 4–5). *p < 0.05.

*CAP exposure increases resident EPCs in bone marrow.* We examined PM_2.5_-induced changes in bone marrow EPCs by culturing cells from the bone marrow on fibronectin-coated plates for 7 days. Nearly 90% of outgrowing spindle-shaped cells were positive for acLDL uptake and UE-lectin binding (Figure 3A). After 10 days in culture, these cells formed tube-like structures (Figure 3B) when seeded on Matrigel, indicating that they had differentiated into endothelial cells. To determine the effects of PM_2.5_ on this cell population, we exposed mice to CAP for 9 days and measured bone marrow Flk-1^+^/Sca-1^+^ cells by flow cytometry (Figure 3C). We found that in contrast to decreased EPC levels in blood, CAP-exposure led to a significant increase in the number of resident EPCs in bone marrow (Figure 3D).

**Figure 4:**
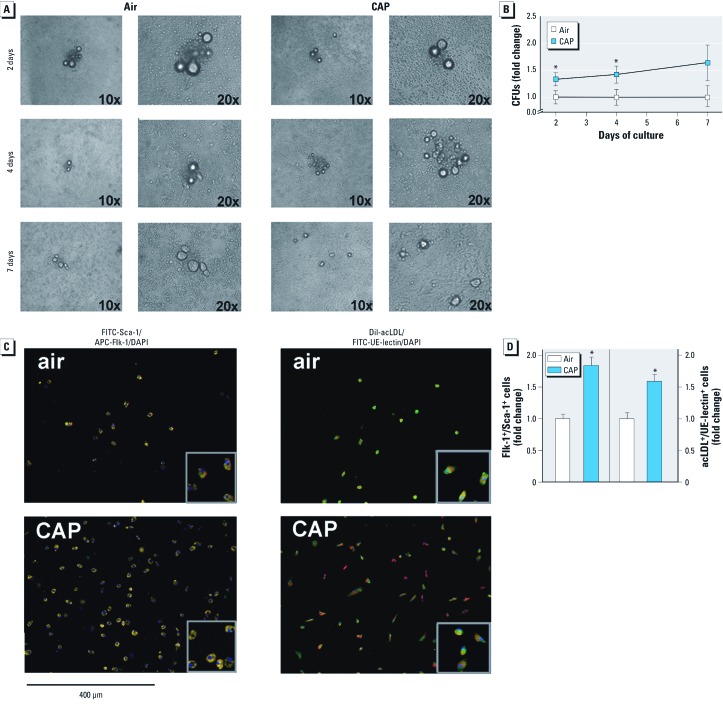
CAP exposure enhances the outgrowth of BMDCs isolated from mice exposed to air or CAP for 9 days. Phase contrast images (*A*) and quantification (*B*) of counted CFUs (fold change) after 2, 4, and 7 days of culture of BMDCs. (*C*) Fluorescence images of BMDCs labeled with FITC-Sca‑1/APC-Flk‑1 or DiI-acLDL/FITC-UE-lectin and DAPI after 7 days of culture. (*D*) Group data of the number of Flk‑1^+^/Sca‑1^+^ and DiI-acLDL^+^/UE-lectin^+^ BMDCs. Data for *B* and *D* are presented as fold change of air control (mean ± SE; *n* = 4). *p < 0.05.

To confirm this finding, we isolated bone marrow from mice exposed for 9 days to CAP or filtered air and cultured the cells in fibronectin-coated dishes. Clusters of cells (cluster forming unit; CFU) appeared within 24 hr after seeding and were counted after 2, 4, and 7 days in culture (Figure 4A; typically 5–20 CFU/well). Cultures of bone marrow cells from CAP-exposed mice developed 50% more CFUs than those from mice breathing filtered air (Figure 4B). In both groups, the number of clusters dissipated within 7 days of culture, giving rise to spindle-shaped cells (Figure 4A), which stained positive for Sca-1 and Flk-1, as well as for acLDL and UE-lectin (Figure 4C). The number of Flk-1^+^/Sca-1^+^ cells or acLDL^+^/UE-lectin^+^ cells was greater in the bone marrow from CAP-exposed mice than in those from mice exposed to filtered air (Figure 4D). These data confirmed flow cytometry results (Figure 3D), showing that CAP exposure increases EPC levels in the bone marrow. Thus, suppression of circulating EPCs in CAP-exposed mice does not appear to be a consequence of EPC depletion in the bone marrow but perhaps is due to a defect in the release of EPCs from the bone marrow.

**Figure 5:**
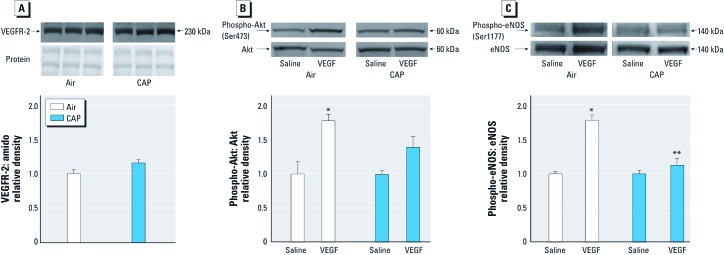
CAP exposure impairs aortic VEGF-signaling. Representative Western blots and densitometric analysis of VEGFR-2 expression (*A*) and VEGF-induced phosphorylation of Akt (*B*) and eNOS (*C*). Western blots were developed from lysates of aortas from mice exposed for 9 days to air or CAP (*A*) and treated with saline or VEGF *ex vivo* (20 ng/mL for 15 min in autologous plasma). Data presented are mean ± SE (*n* = 4–8). *p < 0.05 compared with the air-exposed saline control. **p < 0.05 compared with air + VEGF.

*CAP exposure suppresses VEGF signaling.* The release of progenitor cells from a stem cell niche is a complex multistep process (Heissig et al. 2002; Rafii and Lyden 2003). EPCs are maintained in a quiescent state by contact with the stromal cells of the bone marrow and are mobilized after tissue injury in response to VEGF or stromal-derived factor-1α (SDF-1α). In the bone marrow, VEGF stimulates Akt, and this increases NO production by phosphorylating eNOS (Urbich and Dimmeler 2004). We found that CAP exposure for 9 days did not affect *a*) plasma levels of VEGF or SDF-1α [see Supplemental Material, Table 4 (http://dx.doi.org/10.1289/ehp.1104206)]; *b*) acetylcholine-induced relaxation of the aorta isolated from these mice (see Supplemental Material, Table 5); or *c*) levels of VEGFR2 in the aorta (Figure 5A). VEGF (20 ng/mL for 15 min) stimulated significant increases in both Akt and eNOS phosphorylation in aortas isolated from mice breathing filtered air; however, these responses were abolished in VEGF-stimulated aortas of CAP-exposed mice (Figure 5B,C). From these data, we conclude that CAP exposure impairs vascular VEGF signaling.

**Figure 6:**
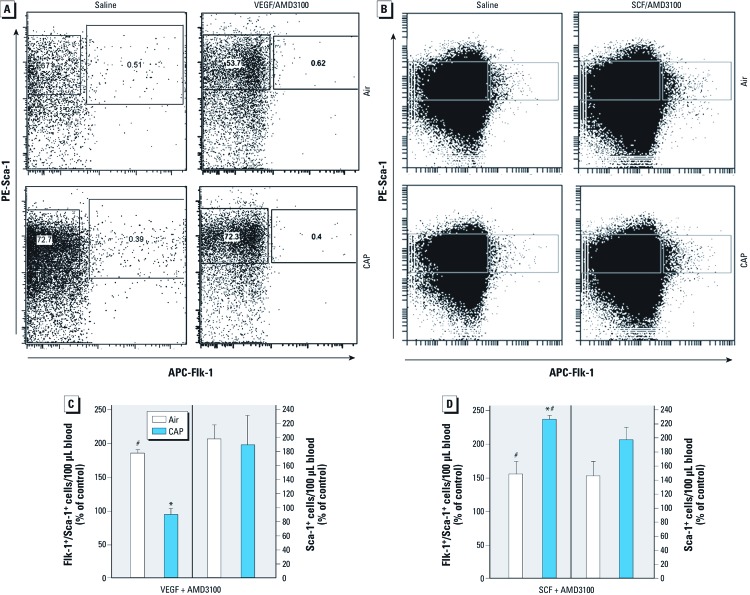
CAP exposure impairs VEGF-induced but not SCF-mediated mobilization of bone marrow Flk‑1^+^/Sca‑1^+^ cells. (*A*) Representative flow cytometry plots of PE-Sca‑1/APC-Flk‑1 immunolabeling of mononuclear blood cells from mice exposed for 9 days to air or CAP and injected with saline or either VEGF/AMD3100 (*A*) or SCF/AMD3100 (*B*). Changes in levels of Flk‑1^+^/Sca‑1^+^ cells or Sca‑1^+^ cells in mice treated with VEGF/AMD3100 (*C*) or SCF/AMD3100 (*D*). Data are presented as a percentage of the saline control (mean ± SE; *n* = 4–9). *p < 0.05 compared with air exposure. ^#^*p* < 0.05 compared with corresponding saline control.

We examined whether VEGF-induced mobilization of EPCs in mice was also affected by 9 days of CAP exposure. On days 6–9 of CAP exposure, mice received daily injections of VEGF; on day 9, 1 hr before euthanasia, mice received a single injection of AMD3100. Treatment with VEGF and AMD3100 (VEGF/AMD3100) led to a 2-fold increase in EPC levels in the blood of saline-injected mice exposed to filtered air; however, no significant increase in circulating levels of EPCs was observed in CAP-exposed mice (Figure 6A,B). Treatment with VEGF/AMD3100 did not affect circulating Sca-1^+^ cells or the CAP-induced retention of EPCs in the bone marrow [see Supplemental Material, Figure 2A,B (http://dx.doi.org/10.1289/ehp.1104206)]. These results indicate that CAP exposure prevents VEGF-induced EPC mobilization from bone marrow to peripheral blood.

Stimulation of VEGFR2 in bone marrow results in MMP-9 activation, which in turn cleaves membrane-bound KitL to generate the soluble KitL (SCF or steel factor) (Heissig et al. 2002). The binding of SCF to c-kit [chemokine receptor on stem cells (CD117)] on stem cells stimulates their mobilization (Thum et al. 2006). We reasoned that if VEGF-induced release of membrane-bound SCF is inhibited but the events downstream of c-kit activation are not affected, then treatment with SCF should rescue CAP-induced EPC suppression. To test this hypothesis, we exposed mice to CAP for 9 days and then treated them with SCF and AMD3100 on the last day of exposure. Flow cytometry revealed that injection of SCF and AMD3100 led to a significant increase in the circulating level of Flk-1^+^/Sca-1^+^ cells (Figure 6C,D) in mice breathing filtered air, and moreover, SCF/AMD3100 treatment led to a significantly greater increase in the number of Flk-1^+^/Sca-1^+^ cells in the blood of CAP-exposed mice. We observed no changes in the circulating levels of Sca-1^+^ cells (Figure 6D) or other blood cells [see Supplemental Material, Table 6 (http://dx.doi.org/10.1289/ehp.1104206)]; however, SCF/AMD3100 treatment also reversed the CAP-induced retention of EPCs in the bone marrow (see Supplemental Material, Figure 2C,D). From these data we conclude that CAP exposure does not affect c-kit signaling and that the CAP-induced decrease in circulating EPC levels could be attributed, in part, to the inhibition of signaling events that are triggered by VEGF/VEGFR2 upstream of c-kit activation.

## Discussion

Although extensive data indicate that PM exposure increases CVD risk, the mechanisms underlying the cardiovascular effects of PM remain obscure (Bhatnagar 2006; Brook et al. 2010). Because PM exposure simultaneously affects multiple aspects of CVD, it is plausible that exposure affects a common determinant of CVD, such as the endothelium. The endothelium is a sensitive target of PM, and animals and humans exposed to PM show endothelial dysfunction (Brook et al. 2011) and EPC depletion (O’Toole et al. 2010), indicating that PM exposure potentially affects the endothelium by acutely causing direct loss of flow-mediated dilation and, in the long term, attenuating EPC-mediated endothelial repair and regeneration. Changes in EPC levels may be particularly informative of the chronic effects of PM and of the mechanisms by which PM exposure increases CVD disease risk.

Current belief is that the levels of circulating EPCs reflect cardiovascular health and that a reduction in EPC levels promotes CVD. Low EPC levels are associated with several CVD risk factors and are predictive of future cardiovascular events (Hill et al. 2003; Vasa et al. 2001). Although the specific role of EPCs in maintaining endothelial function is unclear, it has been suggested that a reduction in EPC levels decreases normal turnover of endothelial cells thereby preventing endothelial repair and regeneration (Steinmetz et al. 2010). Moreover, EPCs promote reendothelialization of arterial lesions, and they suppress smooth muscle cell growth (Friedrich et al. 2006); thus, changes in EPCs could promote hypertension and the development of atherosclerotic plaques. Therefore, EPC depletion may be one mechanism by which PM exposure increases CVD risk.

In agreement with our previous study (O’Toole et al. 2010), we found that CAP exposure decreased EPC levels. In addition, the present data show that CAP exposure leads to an early decrease in circulating levels of EPCs at low levels of PM_2.5_ and that this decrease is reversed when the exposure is withdrawn, suggesting that at least part of the CVD risk imposed by PM exposure may be reversible. This is consistent with epidemiological evidence showing that, in several U.S. cities, a reduction in PM_2.5_ levels increased life expectancy (Pope et al. 2009). A similar reversal of CVD risk has also been reported for smoking cessation (Chelland Campbell et al. 2008). Exposure to tobacco smoke is also associated with changes in EPCs; Heiss et al. (2008) reported that even a brief 30-min exposure to secondhand tobacco smoke impaired VEGF-induced EPC mobilization, although in that case exposure was associated with an increase rather than a decrease in blood EPC levels. Because environmental tobacco smoke is a complex mixture, it is difficult to compare the effects of secondhand smoke to ambient PM; however, our previous studies show that short-term (4-day) exposure to acrolein suppressed EPC levels in mice (Wheat et al. 2011). Similarly, chronic smoking is associated with a profound reduction in circulating EPC levels (Yue et al. 2010) that rebound upon cessation (Kondo et al. 2004), suggesting that a reversible loss of circulating EPCs may be a common response to inhaled pollutants.

Our results indicate that EPC depletion in CAP-exposed mice could not be attributed to an increase in EPC death or excessive tissue recruitment. Instead we found that EPC depletion was associated with VEGF resistance in the aorta and in the bone marrow. These observations suggest that CAP exposure inhibits signaling mechanisms that recruit EPCs from the bone marrow to sites of injury. However, this defect does not appear to be due to a general nonspecific suppressive effect of CAP but a selective inhibition of VEGF-signaling. This notion is supported by the observation that CAP exposure did not affect the mobilization or the circulating levels of stem (Sca-1^+^) cells that do not express VEGF-receptor (Flk-1). Moreover, even though the Sca-1^+^ cells were mobilized from the bone marrow upon CXCR4 stimulation (Figure 2C), their mobilization was unaffected by CAP exposure, indicating that CAP selectively suppress VEGF-sensitive cells. A VEGF-selective effect of CAP exposure is also consistent with our observation that SCF treatment rescued EPC mobilization and reversed bone marrow retention of EPCs.

During EPC mobilization, SCF activation is downstream of VEGF signaling. Thus, our data suggest that CAP exposure inhibits signaling events downstream of VEGF receptor stimulation but upstream of c-kit activation. Interestingly, constituents of tobacco smoke have also been shown to prevent VEGF signaling in endothelial cells (Edirisinghe et al. 2010), and exposure to secondhand smoke prevents VEGF-induced migration of EPCs *ex vivo* (Heiss et al. 2008). Although molecular pathways that mediate pollutant-induced VEGF resistance have not been identified, inhibition of VEGF-signaling by tobacco smoke could be prevented by treatment with antioxidants (Edirisinghe et al. 2010). Hence, it is likely that oxidative stress induced by PM_2.5_ components could be one mechanism underlying this defect. Our chemical analyses have indicated that the elemental composition of Louisville PM_2.5_ is similar to coal fly ash (Smith et al. 2006). It contains high levels of organic carbon, metals, and sulfate, each of which could potentially induce oxidative stress and thereby inhibit VEGF-signaling.

In addition to inhaled pollutants, diabetes also suppresses EPC levels (Fadini and Avogaro 2010); however, the effects of diabetes appear to be different from those of PM_2.5_. In diabetics, circulating levels of CD34^+^ cells are negatively correlated with the percentage of apoptotic CD34^+^ cells, and thus EPC levels are decreased in the peripheral blood due to EPC apoptosis and/or bone marrow retention (Fadini et al. 2010). In contrast, we found that CAP exposure did not trigger EPC apoptosis and that it increased EPC number in the bone marrow. Thus, the mechanism by which PM_2.5_ suppresses circulating EPC levels may be different from that by which diabetes affects EPCs. Further identification of the potentially unique loci of CAP-induced injury will help in distinguishing the effects of PM_2.5_ from the EPC suppressive effects of other insults.

## Conclusion

The present study shows that exposure to ambient PM_2.5_ suppresses EPC levels by preventing their mobilization from the bone marrow due to selective impairment of VEGF-signaling. Based on these data we suggest that suppression of EPC levels may significantly contribute to endothelial dysfunction and excessive CVD risk imposed by long-term PM_2.5_ exposure. Additional studies are required to elucidate the molecular mechanism of this phenomenon, to identify the specific PM_2.5_ component(s) that affect EPC levels, and to assess long-term cardiovascular consequences of air pollution-induced changes in EPC mobilization.

## Supplemental Material

(291 KB) PDF
