# Supplementary material for: Exposure to Ambient Air Fine Particulate Matter Prevents VEGF-Induced Mobilization of Endothelial Progenitor Cells from the Bone Marrow
Source: Environ Health Perspect. 2012 Mar 14;120(6):848–56. doi: 10.1289/ehp.1104206 (PMC3385427; doi:10.1289/ehp.1104206)
Supplement: (291 KB) PDF [file ehp.1104206.s001.pdf]

Supplemental Material

Exposure to Ambient Air Fine Particulate Matter Prevents VEGF-Induced Mobilization of Endothelial Progenitor Cells from the Bone Marrow

Petra Haberzettl, Jongmin Lee, Dheeraj Duggineni, James McCracken, Duane Bolanowski,

Timothy E. O'Toole, Aruni Bhatnagar, Daniel J. Conklin[#]

Diabetes and Obesity Center, Univ. of Louisville, Louisville, KY 40292

***Running head**:* $PM_{2.5}$ inhibits progenitor cell mobilization

[#]Corresponding Author:

Daniel J. Conklin, Ph.D.,
Division of Cardiovascular Medicine,
Department of Medicine,
Delia Baxter Building,
580 S. Preston St., Rm. 404E,
University of Louisville,
Louisville, KY 40202
Tel: (502) 852-5836
Fax: (502) 852-3663
e-mail: dj.conklin@louisville.edu

Supplemental Material: Table of Contents

Page	*Content*
1	Title Page
2	Table of Contents
3-7	Materials and Methods
8	Supplemental Material, Figure 1
9-10	Supplemental Material, Figure 2
11	Supplemental Material, Table 1
12	Supplemental Material, Table 2
13	Supplemental Material, Table 3
14	Supplemental Material, Table 4
15	Supplemental Material, Table 5
16	Supplemental Material, Table 6
17	Supplemental Material, References

Supplemental Material, Materials and Methods:

Ambient $PM_{2.5}$ and concentrated ambient air fine particulate matter (CAP) analysis: All CAP exposures were performed at the Medical and Dental Research building (7th floor) at the Health Science Center, University of Louisville, Louisville, KY (38°15'15''N/85°45'33''W). Ambient $PM_{2.5}$ were concentrated using a Versatile Aerosol Concentration Enrichment System (VACES). A single-stage stainless steel filter holder (URG-2000-30-FV-QCM, URG Inc., Chapel Hill, NC) with teflon filter (47 mm diameter) was used to collect ambient $PM_{2.5}$ or CAP at 10L/min or 1L/min air flow rate, respectively. Collected PM-mass concentrations were determined gravimetrically using a microbalance (XP 26, Mettler-Toledo Inc. Columbus, OH) in a temperature- and humidity-controlled room. The concentration of CAP varied between experiments as a function of ambient $PM_{2.5}$ levels and meteorological conditions including temperature and relative humidity as summarized in the Supplemental Material, Table 1. In addition, a nephelometer (DataRAM 4, Thermo Electron Corporation, Franklin, MA) was used to measure real-time CAP mass concentration and mass median diameter (MMD).

For analysis of ambient $PM_{2.5}$ or CAP chemical composition and physical properties, particles were collected using different filter types (i.e., for specific endpoints: quartz filter: organic carbon (OC), elemental carbon (EC); nylon filter: sulfate, nitrate; teflon filter: ammonium, elements, particle size distribution). The OC and EC compositions were analyzed by a thermal-optical analysis technique (Sunset Laboratory; Portland, OR, USA). Standard US EPA methods were used to analyze sulfate, nitrate (Inter-Mountain Lab, Sheridan, WY, USA) and ammonium ion levels (Columbia Analytical Services, Kelso, WA, USA). Elemental analysis was conducted by X-ray fluorescence spectrometry (XRF; EX-6600-AF, Jordan Valley,

Austin, TX, USA). Ambient $PM_{2.5}$ and CAP showed no significant difference of mass fraction (as %) in chemical or elemental composition (Figure 1A,C; Supplemental Material, Table 2).

Particle size distributions (geometric particle diameter) of ambient $PM_{2.5}$ and CAP were analyzed as geometric particle number distributions using a filter-based particle size analyzer (Malvern Mastersizer 2000, Malvern Instrument Ltd, Worcestershire, UK). To better characterize the relationship between CAP exposure and EPC suppression, exposure duration and cumulative CAP load (µg) were regressed against EPC level. The estimate of CAP load (µg) was calculated according to: CAP (µg/m^3) * chamber flow (m^3/min) * exposure time (min). We estimated lung deposition of PM for each specific exposure using mouse tidal volume, breathing frequency and an estimated deposition fraction. For example, for a 25g mouse with ventilation frequency of 110 bpm, tidal volume of 200 µl and a conservative estimate of $PM_{2.5}$ deposition of 15 % for 9 days (6h/d) to an average CAP of 50 µg/m^3, the estimated total lung CAP burden was <1µg/mouse.

Flow cytometry: Levels of EPCs were measured as described before (O'Toole et al. 2010; Wheat et al. 2011). Briefly, circulating Flk-1$^+$/Sca-1$^+$-cells were analyzed from ~ 400-500 µl whole blood that was treated twice with BD PharmLyse (BD BioSciences, San Jose, CA, USA; 10 min, RT). Bone marrow Flk-1$^+$/Sca-1$^+$-cells were examined after separation of bone marrow aspirates by Ficoll gradient centrifugation (Ficoll-Paque PREMIUM, GE Healthcare, Piscataway, NJ, USA; 400xg, 20 min, 4°C). Cells obtained from blood or bone marrow were incubated with murine CD16/CD32 Fc-Block (0.5 µg; BD Biosciences, 10 min on ice in 1% FBS/PBS) and immuno-labeled by incubation (30min, 4°C) with Sca-1 (PE, 1 µg; BD BioSciences) and Flk-1 (APC, 1 µg, BD BioSciences) antibodies. Appropriate isotype controls (1 µg; BD BioSciences) were used to exclude non-specific binding. The number of Flk-1$^+$/Sca-1$^+$-cells was measured by

flow cytometry using an LSRII flow cytometer (BD BioSciences) and analyzed with FlowJo version 8 software (Treestar software). As shown in Figures 2A&3B, Flk-1$^+$/Sca-1$^+$-cells were electronically-gated based on forward (FSC) and side scatter (SSC), in a small non-debris, sub-lymphocytic population (3-5 µm; cell size was estimated by using size-calibrated beads, Invitrogen, Carlsbad, CA, USA, Wheat et al. 2011) and displayed in a two-color dot plot. From the dot plot, the number of Flk-1$^+$/Sca-1$^+$-cells in blood or bone marrow was measured. Apoptosis in the circulating Flk-1$^+$/Sca-1$^+$-cell population was analyzed by Annexin-V/7-AAD labeling. Mononuclear cells were incubated with Annexin Binding Buffer (100 µl; Annexin Kit, eBioscience, 15 min, RT) followed by an incubation with Fc block and Flk-1 and Sca-1 antibodies as described above. Finally cells were labeled with 7-AAD (5 µl; eBioscience) and analyzed by flow cytometry.

Cell culture: Blood mononuclear cells were cultured from 250 µl lysed blood (≈1-4x10^5 viable cells/chamber) on fibronectin-coated (10 % human fibronectin, Sigma-Aldrich) 8-well chamber slides (BD BioSciences, San Jose, CA, USA) in endothelial basal media (EBM, Clonetics/Lonza) supplemented with 20 % FBS (Invitrogen Carlsbad, CA, USA), human endothelial growth factor (hEGF), hydrocortisone, gentamycin/amphotericin B (GA) and bovine brain extract (BBE; SingleQuot®, Clonetics/Lonza) under standard cell culture conditions (37°C, 5 % CO_2). For culture of bone marrow-derived cells (BMDCs), bone marrow aspirates were separated by Ficoll gradient centrifugation (Ficoll-Paque PREMIUM, GE Healthcare, Piscataway, NJ, USA; 400xg, 20 min, 4°C) and 8x10^5 viable cells were used for culture as described above for circulating cells (Seeger et al. 2005). Cells were cultured for 4 (blood-derived) or 7 (BMDCs) days, and then incubated with DiI-acLDL (2.4 µg/ml, Molecular Probes, Invitrogen, Carlsbad, CA, USA) in media for 3 h. After media removal, cells were washed three times with PBS, fixed in 4 %

PFA/PBS (pH 7.4; RT, 10 min) and incubated with FITC-UE-lectin (50 µg/mL, Sigma-Aldrich) at 37°C for 30 min. To label BMDCs, fixed (4 % PFA/PBS, pH 7.4, RT, 10 min) cells were incubated with FITC-Sca-1 (1:25; BD BioSciences) and APC-Flk-1 (1:15, BD BioSciences) antibodies for 1 h at RT. Cells were washed three times with PBS before slides were mounted in DAPI-containing Slow Fade® Gold anti-fade reagent (Invitrogen). DiI-acLDL/FITC-UE-lectin and FITC-Sca-1/APC-Flk-1 double-positive cells were then counted in 10 random microscopic fields ($EVOS_{fl}$, AMG, Hill Creek, WA).

Plasma analysis: Plasma from mice exposed for 9 days to air or CAP was stored at -80 °C until assayed for VEGF and SDF-1α using murine-specific ELISA kits from R&D Systems, Inc. (Minneapolis, MN) following manufacturer's instructions.

Isolated aorta studies: Thoracic aortas were isolated for assessment of either VEGF signaling or vascular reactivity as described previously (Wheat et al. 2011; Conklin et al. 2009) with some modifications.

VEGF signaling. Thoracic *a*ortas from distal of aortic arch to the diaphragm was isolated from mice exposed for 9 days to air or CAP, cleaned in cold PBS, and then placed in autologous plasma for 1 h at 37°C prior to addition of saline (vehicle) or VEGF (20ng/ml, 15 min). After incubation, aortas were snap frozen in liquid nitrogen and stored at -80 °C before use for Western blotting described below.

Vascular reactivity. Briefly, one 3-4-mm aortic ring per mouse was hung on stainless steel hooks in 15-ml water-jacketed organ baths in physiological salt solution (PSS) bubbled with 95% O_2 and 5% CO_2 at 37°C. The composition of PSS was (in mM): NaCl, 119; KCl, 4.7; $MgSO_4 \cdot 7H_2O$, 1.2; KH_2PO_4, 1.2; $NaHCO_3$, 25; $CaCl_2$, 1.6; glucose, 11.1; pH 7.4 (Lohn et al. 2002). Aortic rings (≈1 g loading tension) were contracted with 100 mM potassium solution (2

seaprate times) and re-equilibrated to ≈1g over 2h before measuring endothelial function. To measure endothelium-dependent relaxation, phenylephrine-precontracted (PE; 10 μM) aorta were relaxed with cumulative concentrations of acetylcholine (ACh; 0.1 nM-10 μM). To measure endothelium-independent relaxation, aortas were precontracted with either 100 mM potassium (9-day study) or with U46619 (thromboxane A_2 analog, 0.1 μM; 30-day study) and then relaxed with cumulative concentrations of sodium nitroprusside (SNP; 0.1 nM-10 μM). Relaxation was calculated as a percentage reduction of agonist-induced tension. The effective concentration producing 50% response (EC_{50}) was assessed by normalizing cumulative concentration responses to 100%, plotting the response vs. the log $[\text{molar}]_{\text{agonist}}$, and then interpolating the EC_{50}. The pD_2 represents the –log(EC_{50}).

Western blot analyses: Heart, lung, spleen and pulverized aortas were lysed in RIPA buffer (50mM Tris•HCl, pH 7.4, 150mM NaCl, 1mM EDTA, 0.25% sodium desoxycholate,1% NP-40, 1:100 protease inhibitor cocktail, 1:100 phosphatase inhibitor), sonicated and centrifuged (13,000xg, 15 min, 4° C). Proteins were separated by SDS-PAGE and transferred to PVDF membranes (Bio-Rad, Hercules, CA, USA) as described previously (Wheat et al. 2011). Membranes were probed with commercially available antibodies against VEGFR-2, phospho-Akt (Ser473), Akt, phospho-eNOS (Ser1177), eNOS (1:1000; Cell Signaling Technology, Danvers, MA, USA) or Id-1 (1:1000, Proteintech, IL, USA) and developed using ECL® plus reagent (Amersham Biosciences, Piscataway, NJ, USA). Detected band intensities (Typhoon 9400 variable mode imager, Amersham Biosciences, Piscataway, NJ, USA) were quantified using Image Quant TL software (Amersham Biosciences).

Supplemental Material, Figures and Legends:

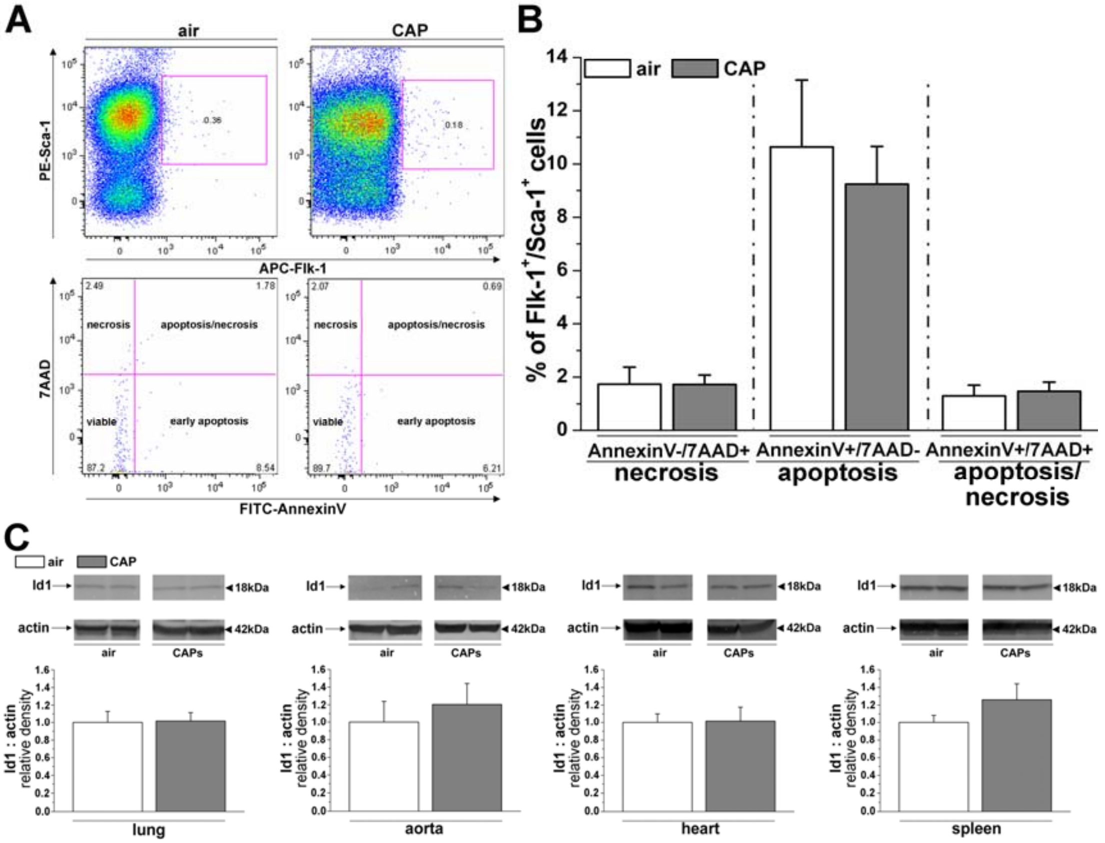

Supplemental Material, Figure 1: Effect of CAP exposure on cell death and tissue homing. (A) Representative flow cytometry plots for measurements of apoptosis and necrosis in the Flk-1^+/Sca-1^+-population. Gated PE-Sca-1^+/APC-Flk-1^+ mononuclear blood cells (upper panel, % gated events) were analyzed for early apoptosis (Annexin-V^+/$7AAD^-$, % apoptotic cells), necrosis (Annexin-V^-/$7AAD^+$, % necrotic cells) or apoptosis/necrosis (Annexin-V^+/$7AAD^+$, % apoptotic/necrotic cells from Flk-1^+/Sca-1^+-cells; lower panel). (B) Group data for Annexin-V^+/$7AAD^-$, Annexin-V^-/$7AAD^+$ or Annexin-V^+/$7AAD^+$-cells within Flk-1^+/Sca-1^+-cells population in PB of mice exposed for 30 days to air or CAP. (C) Representative Western blots and densitometric analysis of lysates prepared from lung, aorta, heart and spleen isolated from mice exposed for 9 days to air or CAP. Western blots were probed with antibodies against Id-1 and actin (loading control). Data are mean±SEM (n=5-8).

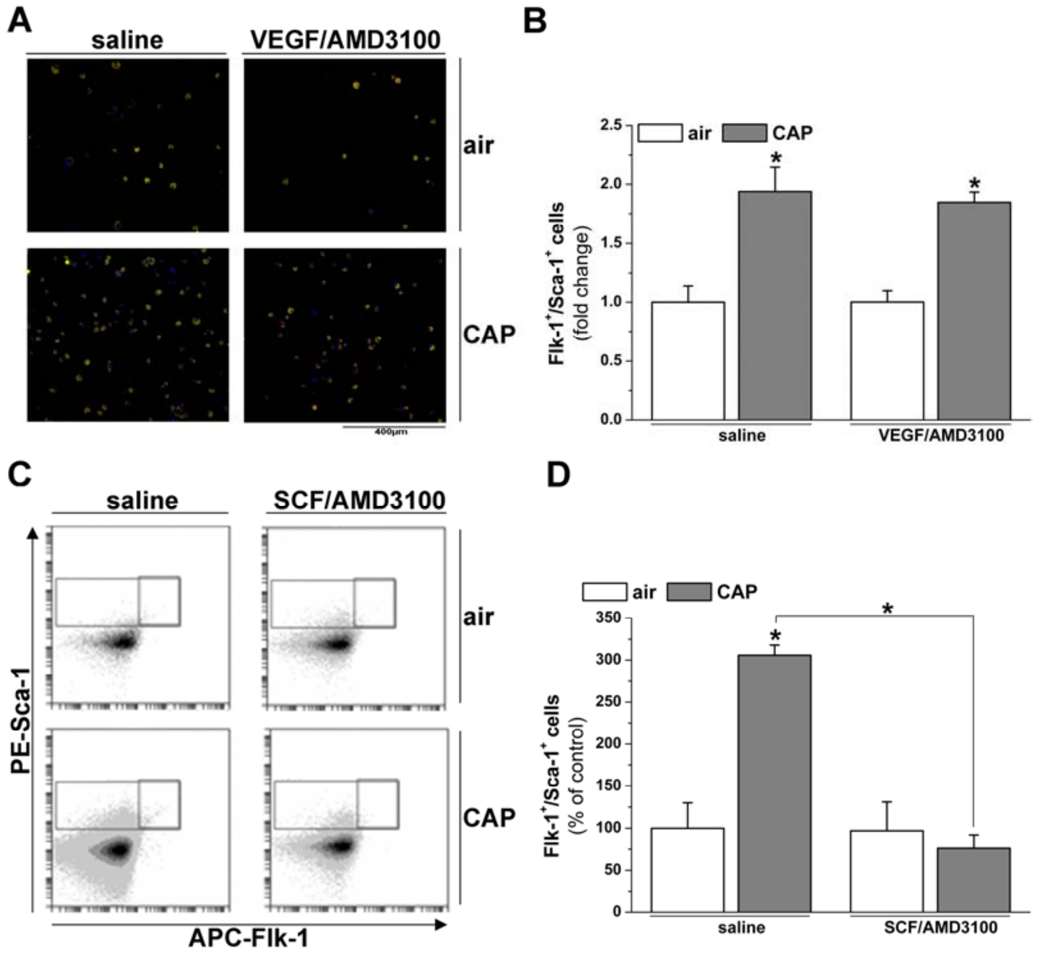

Supplemental Material, Figure 2: CAP exposure impairs VEGF-induced but not SCF-mediated mobilization of bone marrow Flk-1$^+$/Sca-1$^+$-cells. (A) Treatment protocol for the induction of VEGF-induced EPC mobilization. Mice were injected on 4 consecutive days with VEGF at the end of a 9-day air or CAP exposure. After the last 6h exposure, mice injected with VEGF were treated with AMD3100 and euthanized 1h post-injection. (B) Representative fluorescence images of FITC-Sca-1/APC-Flk-1 staining of mononuclear bone marrow cells cultured in fibronectin-coated plates for 7 days from mice exposed for 9 days to air or CAP and injected with saline (vehicle) or the combined VEGF/AMD3100 treatment. (C) Group data of levels of cultured bone marrow Flk-1$^+$/Sca-1$^+$-cells. (D) Protocol for the treatment of mice with SCF and AMD3100. Mice exposed for 9 days to air or CAP received a bolus of SCF and AMD3100 on

the last day of exposure. Representative flow cytometry scatter plots (E) and group data (F) for changes in the number of isolated bone marrow Flk-1^+/Sca-1^+-cells in SCF/AMD3100-treated mice exposed to either filtered air or CAP for 9 days. Data are mean±SEM (* $p<0.05$ air vs. CAP; or as indicated; n= 4-9).

Supplemental Material, Tables:

Supplemental Material, Table 1: Ambient fine particulate matter ($PM_{2.5}$) and concentrated ambient $PM_{2.5}$ (CAP) concentrations for different exposures performed between June 2009 and December 2010. Data indicate VACES $PM_{2.5}$ concentration factors of 2.7-9.3-fold.

Exposure Dates	Duration	$PM_{2.5}$[a]	CAP[a]	Conc. Factor
06-14-09 to 06-22-09	9 days	10.9	101.5	9.3
08-09-10 to 08-17-10[1]	9 days	11.9	106.7	9.0
08-04-10 to 09-03-10[2]	30 days	10.8	87.2	8.1
06-21-10 to 06-29-10[3]	4-9 days	10.8	29.3	2.7
12-07-10 to 12-15-10[4]	9 days	9.1	50.8	5.6
04-21-11 to 04-28-11	9 days	6.9	31.5	4.6

Data are based on gravimetric filter measurements defined as mass divided by air flow (L/min). Unit: [a] $\mu g/m^3$. Note: [1, 2, 3, 4] superscripts are also present in Figure 2F and relate specific CAP exposure with the corresponding EPC level measured in that study.

Supplemental Material, Table 2: Louisville ambient $PM_{2.5}$ elemental content and VACES-induced CAP elemental enrichment.

	$PM_{2..5}$			CAP (enrichment factor)		
Element	2009 June	2010 August	2010 December	2009 June	2010 August	2010 December
S	496	586	325	4324 (8.7)	5192 (8.9)	2007 (6.2)
Si	188	109	72	1745 (9.3)	901 (8.2)	293 (4.1)
Fe	101	136	63	864 (8.6)	682 (5.0)	352 (5.6)
Al	103	58	55	931 (9.1)	571 (9.9)	293 (5.3)
Ca	86	46	34	586 (6.8)	330 (7.1)	179 (5.3)
P	43	28	8	723 (17.0)	233 (8.3)	31 (4.1)
Na	63	27	16	756 (12.0)	104 (3.8)	113 (7.2)
K	49	20	16	535 (10.9)	137 (7.0)	89 (5.5)
Zn	12	6	6	63 (5.2)	45 (7.5)	36 (5.8)
Ti	6	4	4	88 (13.9)	37 (8.9)	16 (4.2)
Cu	8	2	3	85 (11.1)	16 (7.0)	12 (4.5)
Cl	2	7	8	22 (10.0)	80 (11.5)	21 (2.7)
Ba	5	7	1	59 (11.0)	49 (6.6)	3 (3.7)
Se	4	2	1	54 (13.9)	18 (8.4)	7 (5.7)
Mn	1	5	3	11 (11.5)	38 (7.8)	18 (5.7)
Mg	< 0.1	3	14	< 0.1	30 (9.0)	49 (4.5)
Co	< 0.1	3	< 0.1	< 0.1	30 (10.1)	< 0.1
Ni	2	< 0.1	1	14 (8.8)	< 0.1	6 (7.6)
Cr	0.1	1	1	1 (10.0)	2 (1.7)	3 (4.3)
Ga	2	< 0.1	< 0.1	33 (17.1)	< 0.1	< 0.1
Ge	2	< 0.1	< 0.1	23 (11.1)	< 0.1	< 0.1
Br	1	1	1	4 (5.3)	4 (3.8)	4 (5.9)
Sr	< 0.1	1	< 0.1	< 0.1	4 (6.1)	< 0.1
In	2	13	5	12 (7.5)	85 (6.4)	14 (2.7)
Sb	< 0.1	10	< 0.1	< 0.1	102 (9.8)	< 0.1
Cs	10	< 0.1	< 0.1	61 (6.0)	< 0.1	< 0.1
Sc	< 0.1	1	< 0.1	< 0.1	12 (10.4)	< 0.1
As	< 0.1	< 0.1	< 0.1	< 0.1	< 0.1	< 0.1
V	< 0.1	< 0.1	< 0.1	< 0.1	< 0.1	< 0.1
factor				10.2	7.5	5.0

Note: Enrichment factors of CAP elements calculated from gravimetric data from 2009 June, 2010 August and 2010 December exposures were 9.3, 8.1 and 5.6, respectively. Unit: ng/m^3.

Supplemental Material, Table 3: Complete blood count in mice exposed to air or CAP for 9 days, 9days plus 7 days of recovery, or 30 days.

	9 days		9 days + recovery		30 days	
	air	CAP	air	CAP	air	CAP
WBC[a]	3.09±0.48	3.23±0.73	4.17±0.73	3.91±0.61	4.23±0.70	3.66±0.25
NE[a]	0.99±0.22	0.71±0.12	1.05±0.05	0.71±0.08*	0.66±0.08	0.82±0.13
LY[a]	1.97±0.27	2.38±0.59	2.89±0.46	2.95±0.45	3.38±0.63	2.38±0.33
MO[a]	0.11±0.02	0.15±0.03	0.17±0.02	0.16±0.03	0.14±0.03	0.07±0.0
RBC[b]	7.63±0.33	8.47±0.19	8.24±0.33	8.42±0.35	8.20±0.25	8.68±0.57
HCt %	35.23±1.70	37.83±1.04	38.83±1.50	39.00±1.51	37.10±0.79	39.75±2.57
Hb[c]	11.57±1.01	13.23±0.43	12.93±0.27	13.50±0.48	12.56±0.20	13.17±0.96
MCV[d]	46.17±0.86	44.60±0.29	46.63±0.03	46.07±0.33	44.78±0.75	45.80±0.22
MCH[e]	15.17±0.98	15.63±0.20	15.73±0.32	16.03±0.19	14.85±0.51	15.18±0.15
MCHC[c]	32.73±1.62	34.96±0.20	33.67±0.66	34.63±0.44	33.10±0.79	33.10±0.27
RDW %	18.63±0.09	17.07±0.49*	17.60±0.45	17.33±0.50	17.68±0.16	19.02±0.18*
PLT[a]	698±23	713±15	530±30	477±26	1468±16	1297±40*
MPV[d]	3.6±0.1	3.4±0.1	4.5±0.1	4.2±0.1*	3.3±0.1	3.2±0.1*

Mice were exposed to air or CAP for 9 to 30 days (6 h/d), and a group was then allowed to recover for 7 days. Values are mean ± SEM, n = 3-4. Abbreviations: WBC, white blood cells; NE, neutrophils; LY, lymphocytes; MO, monocytes; RBC, red blood cells; HCt, hematocrit; Hb, hemoglobin; MCV, mean corpuscular volume; MCH, mean corpuscular hemoglobin; MCHC, mean corpuscular hemoglobin concentration; RDW, red cell distribution width; PLT, platelets; MPV, mean platelet volume. Units: [a] = [$x10^3$/µL], [b] = [$x10^6$/µL], [c] = [g/dL], [d] = [fL], [e] = [pg]; *, $p<0.05$ vs. air-matched control group.

Supplemental Material, Table 4: Plasma VEGF and SDF-1α levels in mice exposed to air or CAP for 9 days.

	Air	CAP
VEGF[a]	7.09 ± 2.97	8.59 ± 3.10
SDF-1α[a]	523.01 ± 52.57	614.75 ± 32.09

Data are mean±SEM; Unit: [a] pg/mL; n = 10 mice/group.

Supplemental Material, Table 5: Endothelium-dependent and independent relaxations in isolated aorta following 9- or 30-days air or CAP exposure in mice.

	9-day		30-day	
Acetylcholine (ACh)	Air	CAP	Air	CAP
Relaxation (% PE)	-86±5	-76±15	-62±5	-64±7
EC_{50} (nM)	329±188	798±622	552±278	960±398
pD_2	6.66±0.21	6.50±0.33	6.51±0.16	6.31±0.21
Sodium nitroprusside (SNP)				
Relaxation (% Agonist)	-51±2	-50±3	-66±2	-55±6
EC_{50} (nM)	159±8	159±23	100±57	45±5
pD_2	6.80±0.02	6.82±0.07	7.26±0.15	7.37±0.05

Male, 12-14 week old C57BL/6 mice were exposed to HEPA-filtered air or CAP for 9 or 30 consecutive days. Values are means ± SEM. Abbr: PE, phenylephrine-induced contraction; EC_{50} = effective concentration producing 50% response; pD_2, $-\log[EC_{50}]$; SNP-induced relaxations were measured in high potassium-precontracted aorta (9-day study) or in thromboxane A_2 analog-precontracted aorta (U46619; 30-day study) as a percentage decrease in agonist-induced tension. Data are mean±SEM. n=4-8 mice/group.

Supplemental Material, Table 6: Complete blood count in mice exposed to air or CAP for 9 days following control (saline, vehicle), VEGF/AMD3100 or SCF/AMD3100 treatments.

	9 days+ saline		9 days+VEGF/AMD3100		9 days+SCF/AMD3100	
	air	CAP	air	CAP	air	CAP
WBC[a]	2.41±0.34	2.50±0.30	5.35±0.30[#]	7.23±0.67[#]	6.22±0.72[#]	7.52±0.65[#]
NE[a]	0.91±0.17	0.94±0.16	2.04±0.15	2.16±0.23[#]	2.63±0.36[#]	2.88±0.42[#]
LY[a]	1.41±0.20	1.48±0.28	3.15±0.28[#]	4.81±0.49[#]*	2.86±0.19[#]	4.45±0.28[#*]
MO[a]	0.08±0.01	0.07±0.02	0.14±0.02	0.22±0.03[#]*	0.12±0.01	0.18±0.04
RBC[b]	7.85±0.18	8.07±0.20	7.07±0.61	7.47±0.49	8.05±0.28	8.06±0.17
HCt %	35.40±0.78	35.77±0.90	32.16±2.79	33.90±2.29	35.93±1.06	35.26±0.63
Hb[c]	11.59±0.35	12.01±0.36	11.94±0.48	12.68±0.42	11.61±0.33	11.62±0.18
MCV[d]	45.10±0.40	44.32±0.20	45.50±0.43	45.38±0.30	44.73±0.31	43.77±0.18
MCH[e]	14.77±0.31	14.90±0.26	17.30±1.15[#]	17.16±0.81[#]	14.47±0.09	14.41±0.14
MCHC[c]	32.71±0.52	33.58±0.50	38.00±2.52[#]	37.86±1.82	32.33±0.14	32.95±0.16
RDW %	17.77±0.33	17.53±0.31	16.82±0.35	17.20±0.50	17.40±0.24	18.27±0.39
PLT[a]	673±19	625±33	449±86[#]	480±37	634±41	641±23
MPV[d]	4.1±0.2	4.1±0.2	4.2±0.2	4.4±0.1	4.4±0.1	4.3±0.1

Mice were exposed to air or CAP for 9 days (6 h/d), and injected with saline, VEGF/AMD3100 or SCF/3100 as described in material and methods. Values are means ± SEM; n = 6-12; $p<0.05$, * air vs. CAP, # saline vs. VEGF/AMD3100 or SCF/AMD3100. Abbreviations: WBC, white blood cells; NE, neutrophils; LY, lymphocytes; MO, monocytes; RBC, red blood cells; HCt, hematocrit; Hb, hemoglobin; MCV, mean corpuscular volume; MCH, mean corpuscular hemoglobin; MCHC, mean corpuscular hemoglobin concentration; RDW, red cell distribution width; PLT, platelets; MPV, mean platelet volume. Units: [a] = [$x10^3/\mu L$], [b] = [$x10^6/\mu L$], [c] = [g/dL], [d] = [fL], [e] = [pg]; *, *p*<0.05 vs. air-matched group.

Supplemental Material, References:

O'Toole TE, Hellmann J, Wheat L, Haberzettl P, Lee J, Conklin DJ, et al. 2010. Episodic exposure to fine particulate air pollution decreases circulating levels of endothelial progenitor cells. Circ Res 107(2): 200-203.

Wheat LA, Haberzettl P, Hellmann J, Baba SP, Bertke M, Lee J, et al. 2011. Acrolein inhalation prevents vascular endothelial growth factor-induced mobilization of flk-1+/sca-1+ cells in mice. Arterioscler Thromb Vasc Biol 31(7): 1598-1606.

Seeger FH, Haendeler J, Walter DH, Rochwalsky U, Reinhold J, Urbich C, et al. 2005. p38 mitogen-activated protein kinase downregulates endothelial progenitor cells. Circulation 111(9): 1184-1191.

Conklin DJ, Haberzettl P, Prough RA, Bhatnagar A. 2009. Glutathione-S-transferase P protects against endothelial dysfunction induced by exposure to tobacco smoke. AmJPhysiol Heart CircPhysiol 296(5): H1586-H1597.

Lohn M, Dubrovska G, Lauterbach B, Luft FC, Gollasch M, Sharma AM. 2002. Periadventitial fat releases a vascular relaxing factor. FASEB J 16(9): 1057-1063.
